# Surgically assisted rapid palatal expansion with tent screws and a custom-made palatal expander: a case report

**DOI:** 10.1186/s40902-015-0011-7

**Published:** 2015-03-21

**Authors:** Kang-Nam Park, Chang Youn Lee, In Young Park, Jwa Young Kim, Byoungeun Yang

**Affiliations:** 1 Department of Oral and Maxillofacial Surgery, Hallym University Sacred Heart Hospital, Gwanpyeong-ro 170beon-gil, Dongan-gu, Anyang-si, Gyeonggi-do, 431-796 Republic of Korea; 2 Department of Orthodontics, Hallym University Sacred Heart Hospital, Gwanpyeong-ro 170beon-gil, Dongan-gu, Anyang-si, Gyeonggi-do, 431-796 Republic of Korea

**Keywords:** Transverse maxillary deficiency, Surgically Assisted Rapid Palatal Expansion (SARPE), Rapid Palatal Expansion (RPE), Tent screw, Palatal expander

## Abstract

Rapid palatal expansion(RPE) with the tooth-born appliance is not sufficient to apply to the patients with periodontal problem or insufficient tooth anchorage, and it leads to tipping of the anchorage teeth and increasing teeth mobility and root resorption. To avoid these disadvantages, we present the case using palatal screws and custommade palatal expander. A 23-year-old patient underwent surgically assisted rapid maxillary expansion with the Hyrax expansion using 4 tent screws. The study models were used to measure the pre−/−post surgical width of the anterior and posterior dental arches with a digital sliding caliper. In the result, the custom-made palatal expander with 4 tent screws is suitable for delivering a force to the mid-palatal suture expansion. And it is low cost, small sized and simply applied. The results indicated that maxillary expansion with the custom-made palatal anchorage device is predictable and stable technique without significant complications in patients.

## Background

Transverse maxillary deficiency is a relatively common clinical problem in both teenagers and adults. This defect may be associated with sagittal or vertical defects of the upper maxilla and mandible [1]. Transverse maxillary deficiency may contribute to crossed posterior unilateral or bilateral bite, as well as anterior dental crowding and black buccal corridors on smiling [2].

Although rapid palatal expansion(RPE) has been a reliable treatment modality in prepubescent patients, there have been controversies regarding nonsurgical expansion in adults [3]. Surgically assisted rapid palatal expansion (SARPE) has been the treatment of choice to resolve the high resistance from the bony palate and the zygomatic buttress [4,5]. The difficulties in treating the transverse discrepancy are associated with the limited range of tooth movement in the transverse dimension [6].

SARPE was traditionally performed with the use of tooth-borne orthodontic devices (Hyrax type, Haas type). Force from the Hyrax expansion screw applied to the molars and premolars results not in a parallel but rather a tipping movement of the right and left maxilla. Additionally, root resorption, gingival recession and pathologic loss of buccal cortical bone and the anchorage teeth can occur. The dental anchorage quality may also be reduced if the maxillary sinus extends far downwards, and there are missing teeth or periodontitis [7,8].

Beacause of many drawbacks and questionable effects of conventional palatal expander that are either tooth-borne or tooth-and-tissue-borne, the bone-borne maxillary distractors have been developed to deliver the expansion force directly to the basal bone and to overcome the dental complications. These appliances reduce disadvantages and produce more predictable results [5,7-9]. However, they are more expensive and cumbersome than conventional orthodontic devices.

To make up for weak points, a custom-made bone-borne appliance was newly designed in a bid to lessen volume of the appliance and to cut costs. We present our experience using a novel palatal expander in an adult patient with closed midpalatal suture.

## Case presentation

**Figure 1:**
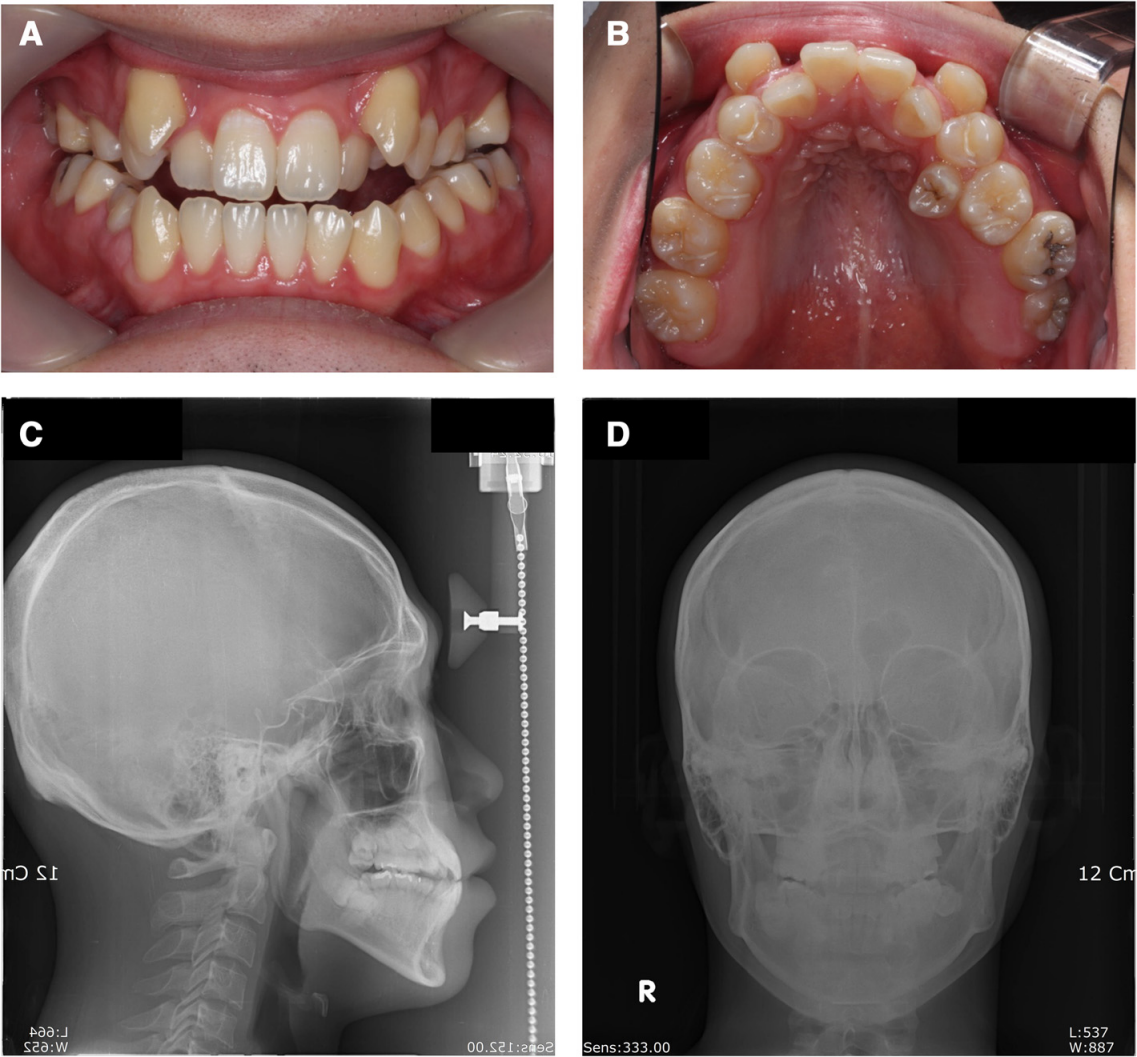
**Pre-treatment intraoral photographs showing transverse maxillary deficiency and posterior crossbite. A**. Frontal view **B**. Occlusal view **C**. Cephalogram(lateral) **D**. Cephalogram(PA).

A 23-year-old man visited with the chief complaint of malocclusion. He was diagnosed with transverse maxillary deficiency and posterior crossbite, which presented a skeletal class I pattern, anterior crowding, labioversion of the incisor teeth, and impaction of the mandibular right second molar by clinical and radiographic examination (Figure 1) The patient had no syndrome and no other medical conditions for transverse maxillary deficiency.

In this case, the patient had closed midpalatal suture, so possible treatment include SARPE and segmented osteotomy of the maxilla. SARPE and osteotomy were planned to correct the transverse compression and posterior crossbite.

**Figure 2:**
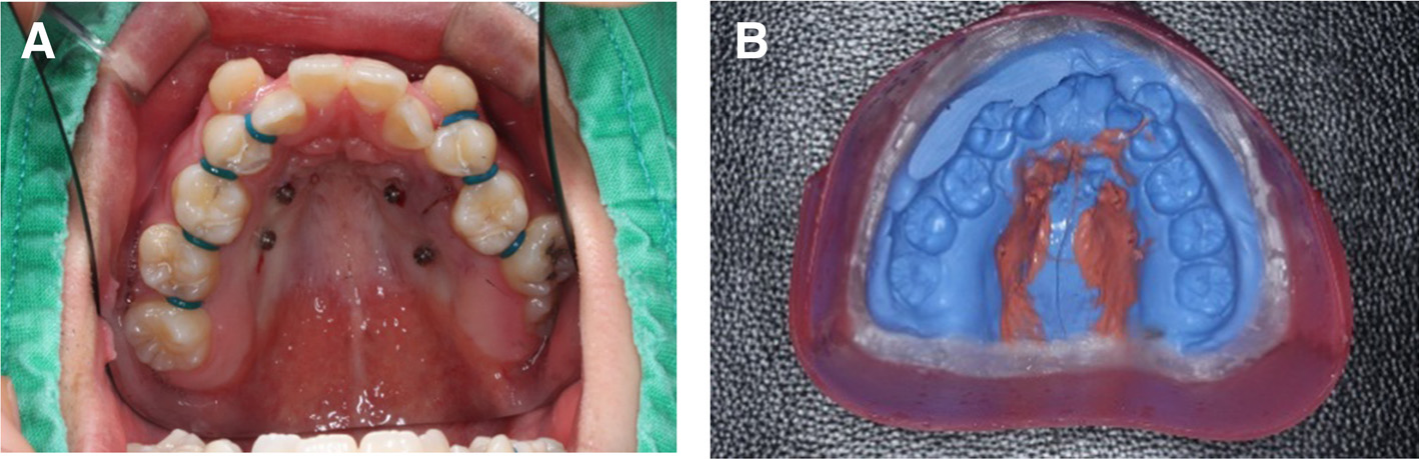
**Tent screws for expansion.** **A.** Four tent screws were inserted prior to the osteotomy surgery under local anesthesia. **B**. Impression of the upper maxillary arch was taken for the appliance.

Four tent screws (Tent screw, Neobiotech, Seoul, South Korea) were placed under local anesthesia prior to the osteotomy surgery, and an impression of the upper maxillary arch was taken for the appliance (Figure 2).

**Figure 3:**
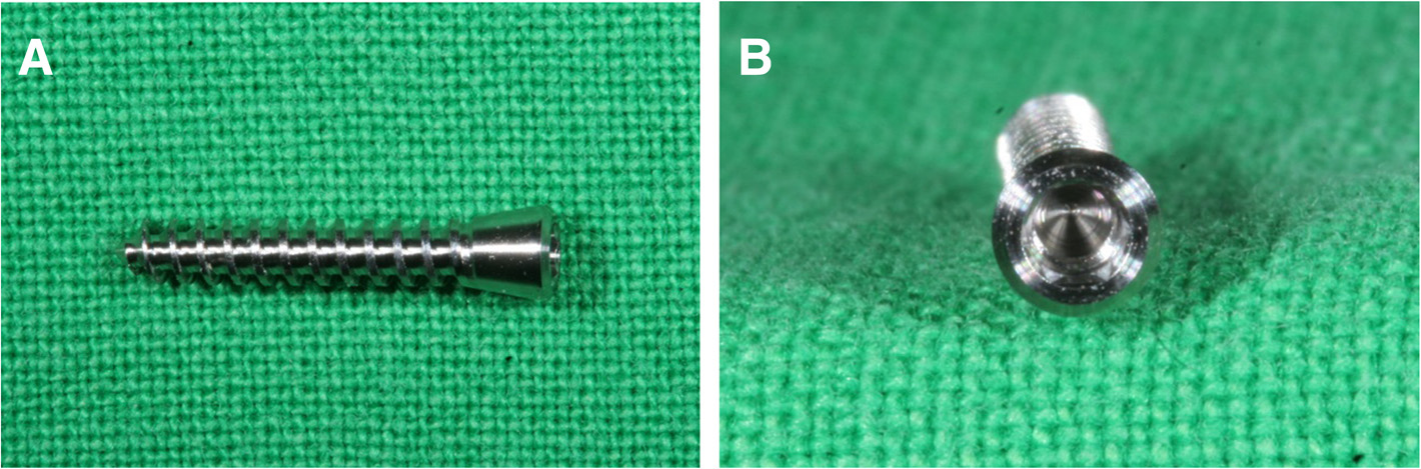
**Tent screw(Tent screw, Neobiotech, Seoul, Korea). A**. Lateral view. **B**. Occlusal View. Tent screw has a hole for the cover screw.

The tent screws had the following dimensions: diameter 2 mm, length 10 mm. Generally, tent screws are used for guided bone regeneration to maintain the space between the membrane and bone for bone formation to ease the fixing of membrane. These screws have a hole for the cover screw, and we used this hole for setting the appliance (Figure 3).

The patient underwent a bilateral osteotomy and splitting of the midpalatal suture according to the procedure described by Glassmann [9]. This procedure was performed under general anesthesia.

**Figure 4:**
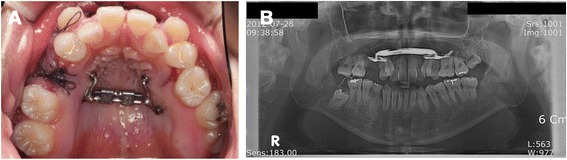
**Adaptation of the customized palatal expander. A.** The patient underwent a bilateral osteotomy and splitting of the midpalatal suture and the appliance was placed on the palatal vault under general anesthesia. **B.** panoramic view after surgery.

The rigid arms of the customized RPE appliance were designed to fit in the cover screw hole, which were made to be 1 mm in the actively expanded state in the laboratory. When the appliance was set in the upper arch, the activated screw was unwound to the inactivated state, placed to fit in the hole and activated to 1 mm again to retain the appliance. So, we could solve the problem if there were some errors on device, and the device could be fitted well on proper position (Figure 4).

**Figure 5:**
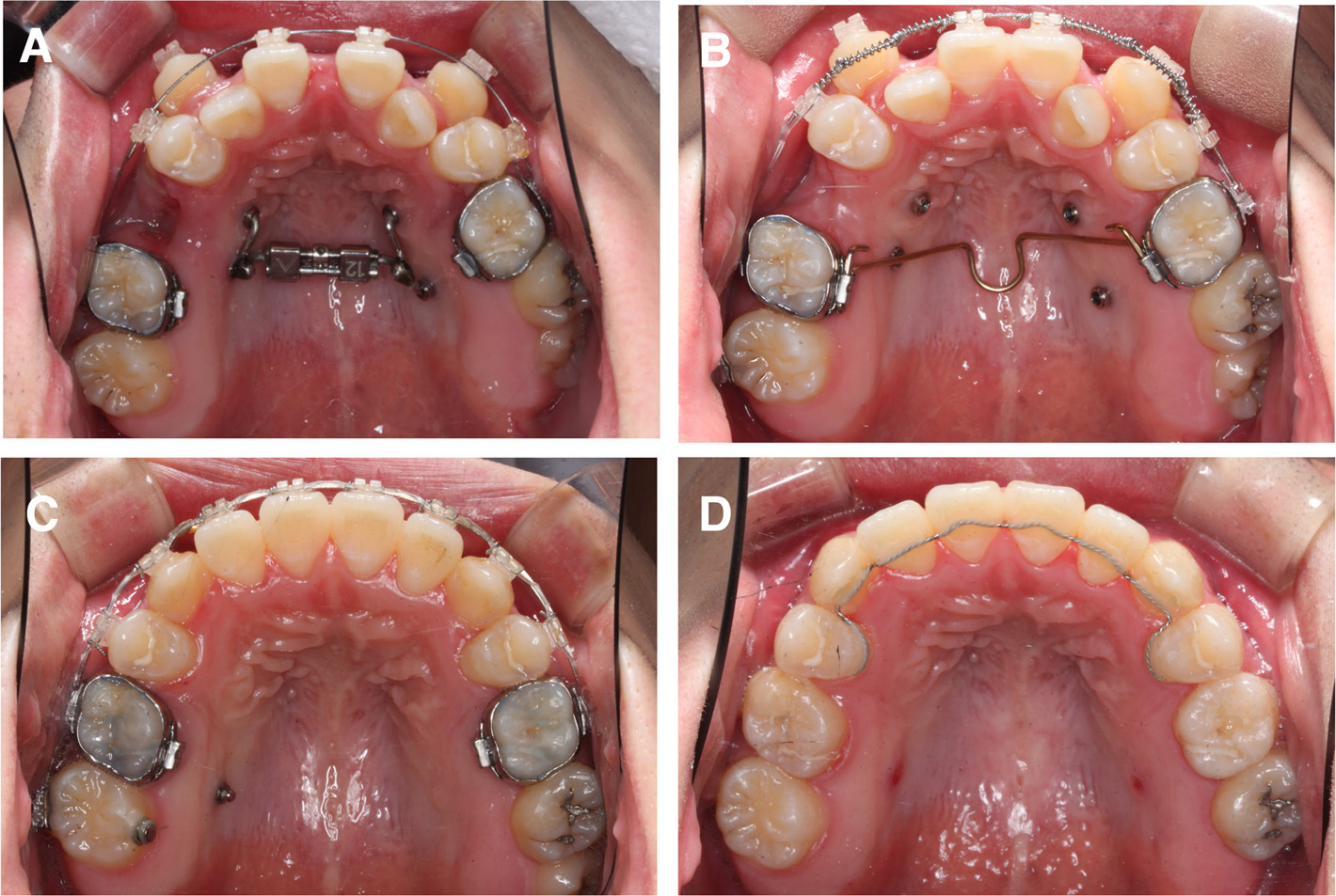
**The appliance was activated 2 times a day (1 activation = + 0.25 mm) for 14 days for a total of 7 mm.** After expansion was complete and the screw was immobilized, the appliance acted as a fixed retainer for a period of 6 months. **A**. 2 weeks After surgery **B**. 6 months after surgery. Palatal expander was removed and TPA appliance was placed for retention. **C**. 1 years after surgery **D**. 2 years after surgery.

We activated the appliance 2 times a day (1 activation = +0.25 mm) for 14 days, for a total of 7 mm. After the expansion was completed and the screw was immobilized, the appliance acted as a fixed retainer for a period of 6 months to allow the tissues to reorganize in their new positions. Orthodontic brackets were placed and orthodontic force was applied after activation was terminated (Figure 5).

Upper arch impressions were taken to include the occlusal surfaces of the teeth using rapid-setting alginate at the time of appliance fitting and after 2 weeks. Using digital calipers, the arch widths were measured on the resulting models between the maxillary first molars and the incisors, and a 2.27-mm increase in the intermolar width and a 3.25-mm increase in the interincisor width were observed after active expansion. This result remained stable after 6 months, 1 years, and 2 years. No significant differences was found. The patient didn’t show dental problem and anchorage loss. Also, the patient felt comfortable on the distraction procedure.

## Discussion

Placing a bone-borne RPE appliance using tent screws is a minimally invasive procedure, and the structure is simple and extremely small. The appliance is a simple modification of the conventional RPE appliance.

This method eliminates risks to the anchorage teeth, such as root resorption and fenestration of the buccal cortical bone, and it allows parallel (bodily) movement. The screws were left in place for the retention. This small appliance offers the patient greater freedom of tongue movement when speaking and eating than traditional palatal expanders. Additionally, there is no need for an additional method to fix the appliance because the rigid arms of the appliance were actively inserted into the tent screw holes. Orthodontic force is allowed during the consolidation period, resulting in the reduction of treatment time.

Expansion arches cost substantially less than distraction osteogenesis appliances. The four-point fixation of the expansion screw can provide sufficient guidance stability for the two halves of the maxilla during expansion. This technique can be advantageously applied to patients without presurgical osteotomy and to children and adolescents. This technique is contraindicated in patients in whom the palatal vault is too low, potentially leading to root tip injury and sinus perforation after screw insertion.

The treatment of transverse maxillary discrepancy in adults using the classical orthodontic transverse expansion technique was ineffective because of the high frequency of complications [2,5,10]: dental rotations, radicular reabsorption, periodontal damage and high recurrence rates of defect after expansion. These complications occurred due to the expansion force that was exerted mainly on the teeth and inability to overcome maxillary osseous suture resistance. Because of these disadvantages, bone-borne maxillary distraction was planned in many cases. Bone-borne palatal expanders reduce the risk of dental damage and provide more predictable results, but ready-made bone-borne RPE appliances are expensive and bulky and are restricted in specific cases.

Phillips et al. reported considerable relapse after expansion with Le Fort I and segmental osteotomies in 39 patients. SARPE, therefore, was expected to show superior stability compared with segmental osteotomy because it allows tissue adaptation during the expansion and subsequent consolidation periods [11].

In this case, the patient had a closed midpalatal suture and dense cortical bone, as observed from computer tomography, and he wished to achieve fast results. Therefore, we planned SARPE with a newly designed custom-made palatal expander using tent screws, and this case showed many advantages in the successful adaptation and function for the treatment of transverse maxillary deficiency.

## Conclusion

The newly developed appliance can be applied to diverse cases by modifying the number and position of the screws, and we can make patients more comfortable and predict stable results.

## Consent

Written informed consent was obtained from the patient for the publication of this report and any accompanying images.

## Endnote

We tried to find a solution for transverse maxillary deficiency and we used palatal expander several times, but tooth-borne palatal expander and ready-made palatal expander has many drawbacks. So, we designed the appliance newly by using tent screws and got a good result. We want to present our experience.

## Abbreviations

RPE: Rapid palatal expansion
SARPE: Surgically assisted rapid palatal expansion
